# Efficacy and safety of apical access in percutaneous pericardiocentesis: a comparison with subxiphoid approach

**DOI:** 10.2459/JCM.0000000000001766

**Published:** 2025-08-20

**Authors:** Marco Biolcati, Andrea Mauro, Francesca Del Furia, Davide Carlo Corsi, Elena Tassistro, Maddalena Lettino

**Affiliations:** a School of Medicine and Surgery, University of Milano–Bicocca, Milan; b Department for Cardiac, Thoracic and Vascular Diseases; c Biostatistics and Clinical Epidemiology, IRCCS San Gerardo dei Tintori foundation; d Bicocca Centre of Bioinformatics, Biostatistics and Bioimaging (B4 centre), School of Medicine and Surgery, University of Milano–Bicocca, Monza, Italy

**Keywords:** apical, pericardial effusion, pericardiocentesis, subxiphoid

## Abstract

**Aim:**

Percutaneous pericardiocentesis represents the sole curative intervention for significant pericardial effusion, especially in cardiac tamponade. While the subxiphoid route is traditionally the most utilized, alternative approaches – such as the apical access – have also been adopted. To date, no studies have directly compared the performance, risk profile, and clinical implications of these techniques. This study aims to evaluate and compare the effectiveness, complication rates, and short- to medium-term outcomes of apical versus subxiphoid pericardiocentesis.

**Materials and methods:**

We performed a retrospective analysis of pericardiocentesis procedures carried out at the Cardiac Intensive Care Unit of the IRCCS San Gerardo dei Tintori Foundation in Monza, Italy, between January 2011 and December 2024. Patients were categorized based on the access site: apical or subxiphoid.

**Results:**

Among 199 procedures, 85 (42.7%) were performed via the subxiphoid route and 114 (57.3%) through apical access. Most interventions addressed acute tamponade or pretamponade states. Imaging guidance was employed in 89.6% of cases. Baseline demographics, comorbidities, and echocardiographic features were comparable between the two groups. The overall success rate was 98.5%, with no significant differences between approaches. Major complications were rare (0.5%), and minor complications occurred in 11.1% of cases, without notable variance between techniques. Patient outcomes – including overall survival, in-hospital survival, and pericardiocentesis-free survival – showed no statistically significant differences (median follow-up: 17.2 months; interquartile range: 3.8–69.2 months).

**Conclusions:**

Apical access for percutaneous pericardiocentesis demonstrates similar efficacy and safety to the subxiphoid approach, representing a valid alternative in appropriate clinical contexts.

## Graphical abstract

**Figure:**
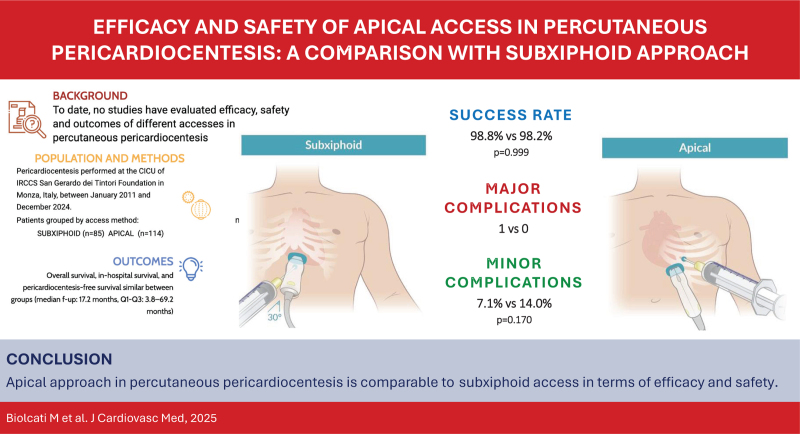

## Introduction

Pharmacological therapy plays a role in the management of pericardial effusion, particularly when the underlying etiology is inflammatory. In such cases, notably pericarditis, the use of anti-inflammatory agents has been well validated and is associated with a progressive reduction in effusion size, often culminating in complete resolution.^1^

Nevertheless, in instances of persistent symptomatic pericardial effusion despite the commencement of pharmacological interventions, definitive treatment is represented by pericardiocentesis. This procedure should be performed emergently, guided by the clinical presentation, particularly in cases of cardiac tamponade.

Three primary entry sites can be employed for accessing the pericardial sac with a needle: apical, subcostal (or subxiphoid), and parasternal. Each access point has its respective advantages and disadvantages, primarily determined by the associated risk of damage to the surrounding structures.^2^

Traditionally, the subxiphoid approach has been favored by operators, as it is considered the safest route in the absence of imaging guidance. However, pericardial effusion is not always circumferential or uniformly distributed, making it essential to utilize ultrasound to assess the optimal entry site for drainage. The Mayo Clinic recommends selecting the most appropriate approach based exclusively on echocardiographic findings, defining the optimal entry site as the point where the pericardial space is closest to the ultrasound (US) probe and fluid accumulation is maximized, with no vital organs interposed. According to these criteria, the para-apical site is more commonly identified as the ideal access point than the subxiphoid approach. Furthermore, observational studies in percutaneous pericardiocentesis have demonstrated higher success rates and lower complication rates when the entry site is selected based on US-guidance.^2–4^

Currently, the literature does not provide comparative studies on the different approaches to percutaneous pericardiocentesis in an urgent clinical setting. This study aims to compare the efficacy, safety, and short- to medium-term outcomes of the apical approach versus the subxiphoid approach in a cohort of patients with pericardial effusion undergoing percutaneous pericardiocentesis.

## Methods

### Patients and setting

A retrospective analysis was conducted on data from all patients who underwent percutaneous pericardiocentesis and were subsequently admitted to the Cardiology Intensive Care Unit (CICU) of the IRCCS San Gerardo dei Tintori Foundation in Monza, Italy, between January 2011 and December 2024. The study cohort was divided into two groups based on the approach used during the procedure (subxiphoid and apical).

Data regarding general and clinical characteristics, procedural details, and relevant outcomes were collected. For all pericardiocentesis procedures, 2D cardiac US data were recorded. Given the time constraints during emergency situations, the echocardiographic evaluation was necessarily abbreviated, and while the exact dimensions of the maximal fluid collection were not documented for all pericardial effusions, the severity range was recorded.

Outcomes of interest included procedural success, major and minor complications, recurrence of pericardial effusion, the need for a second pericardiocentesis, and survival. For the latter three outcomes, both events occurring during the index hospitalization and any subsequent outpatient visits or hospitalizations were included.

Percutaneous pericardiocentesis was considered successful if it resulted in a significant reduction of the pericardial space (defined as at least an 80% decrease in size) and resolution of signs and symptoms of cardiac tamponade, when present.

Major complications were defined as any adverse events related to the procedure that led to death or necessitated a serious intervention (including surgery or other interventional procedures) due to hemodynamic instability or significant organ damage. This category included laceration of intercostal vessels, puncture of abdominal viscera or the peritoneal cavity, pneumothorax requiring chest drain placement, pneumopericardium, sustained ventricular arrhythmias, and pericardial decompression syndrome.

Minor complications encompassed those events that did not require intervention beyond appropriate monitoring and follow-up, such as transient vasovagal hypotension and bradycardia, supraventricular arrhythmias, pneumothorax without hemodynamic instability, and pleuro-pericardial fistulas.

Recurrences were defined as the presence of similar or larger effusions than previously recorded in any subsequent echocardiographic examination.

### Choice of the entry site

All the pericardiocenteses were conducted by medical personnel with established and comparable experience in the technique, predominantly consisting of CICU physicians for bedside procedures or interventional cardiologists for drainages performed within the cath lab.

The criteria used to select the entry site between apical and subxiphoid included the location of the maximal amount of the fluid layer, as identified through preliminary echocardiographic analysis, and the most optimal needle trajectory at the intra-abdominal and intra-thoracic levels, with the objective of minimizing potential damage to adjacent organs. However, it is important to highlight the presence of a certain degree of inter-operator variability in the selection process, influenced by individual experience and familiarity with one approach over the other.

### Statistical analyses

The characteristics of the cohort, both for the entire sample and stratified by the type of approach used, were described using the mean and standard deviation (SD) or the median with the first (Q1) and third (Q3) quartiles, after verifying the normality of the distribution for continuous variables. Absolute frequencies and percentages were employed to describe categorical variables.

Univariate analyses were performed using the *t*-test or the Wilcoxon test, as appropriate, for continuous variables, and the chi-square test or Fisher's exact test for categorical variables. The Kaplan–Meier nonparametric estimator was used to calculate overall survival (including deaths during both the index hospitalization and subsequent admissions), in-hospital survival, recurrence-free survival for pericardial effusion, and survival free from subsequent pericardiocentesis in the entire population and across the two groups stratified by the approach type. The survival curves between the two groups were compared using the log-rank test.

All analyses were conducted using the statistical software R, version 4.4.1 (available at http://cran.r-project.org/). *P*-values were two-tailed, with *P*-values <0.05 considered statistically significant.

## Results

A total of 199 consecutive pericardiocentesis procedures were performed over a 14-year period. Of these, 85 (42.7%) were conducted via subxiphoid access, while 114 (57.3%) were performed using an apical approach.

Table 1 outlines the baseline characteristics of the cohort, with clinical presentation and comorbidities appearing largely comparable between the two groups. More than half of the procedures were carried out in patients with acute cardiac tamponade. Only 7% of the cases involved symptomatic pericardial effusions despite ongoing therapy (mostly anti-inflammatory medications in cases of pericarditis complicated by significant effusion) or the sampling of cardiac fluid for diagnostic purposes.

**Table 1:** Baseline characteristics of the population

	Total (*N* = 199)	Subxiphoid approach (*N* = 85, 42.7%)	Apical approach (*N* = 114, 57.3%)	*P*-value
Women, *N* (%)	69 (34.7)	30 (35.3)	39 (34.2)	0.993
Age (years), median (Q1–Q3)	70.4 (57.2–77.4)	69.7 (60.2–76.6)	70.8 (55.9–77.9)	0.915
Clinical presentation, *N* (%)				0.069
*Cardiac tamponade*	103 (51.8)	51 (60.0)	52 (45.6)	
*Precardiac tamponade*	82 (41.2)	31 (36.5)	51 (44.7)	
*Pericardial effusion without cardiac tamponade*	14 (7.0)	3 (3.5)	11 (9.6)	
Known pericardial effusion, *N* (%)	52 (26.1)	12 (14.1)	40 (35.1)	0.002
Prior pericardiocentesis, *N* (%)	4 (2.0)	2 (2.4)	2 (1.8)	0.999
Comorbidities, *N* (%)				
*Hypertansion*	118 (59.3)	55 (64.7)	63 (55.3)	0.232
*Dislipidemia*	61 (30.7)	25 (29.4)	36 (31.6)	0.863
*Diabetes mellitus*	26 (13.1)	10 (11.8)	16 (14.0)	0.797
*Coronary artery disease*	27 (13.6)	16 (18.8)	11 (9.6)	0.097
*Atrial fibrillation*	76 (38.2)	34 (40.0)	42 (36.8)	0.760
*Heart failure*	24 (12.1)	7 (8.2)	17 (14.9)	0.226
*COPD*	14 (7.0)	5 (5.9)	9 (7.9)	0.788
*Anemia*	103 (51.8)	39 (45.9)	64 (56.19)	0.197
*CKD*	67 (33.7)	26 (30.6)	41 (36.0)	0.521
*Active or prior cancer*	75 (37.7)	28 (32.9)	47 (41.2)	0.459
≥3 comorbidities, *N* (%)	107 (53.8)	43 (50.6)	64 (56.1)	0.527
LVEF, median (Q1–Q3)	58.0 (54.0–62.0)	58.0 (53.0–61.5)	58.0 (55.0–62.0)	0.683
Size of the effusion, *N* (%)				0.001
Mild	11 (6.2)	10 (14.1)	1 (0.9)	
Moderate	56 (31.6)	23 (32.4)	33 (31.1)	
Severe	110 (62.1)	38 (53.5)	72 (67.9)	
Maximum size of the effusion (mm), median (Q1–Q3)	25.0 (18.5–30.0)	24.0 (15.0–30.0)	27.0 (20.0–32.5)	0.079
Distribution of the maximum thickness of the effusion, *N* (%)				0.002
Ubiquitariy	135 (67.8)	60 (70.6)	74 (65.8)	
Left ventricle	20 (10.1)	13 (15.3)	7 (6.1)	
Inferior wall	6 (3.0)	5 (5.9)	1 (0.9)	
Anterior wall	1 (0.5)	–	1 (0.9)	
Lateral wall	22 (11.1)	5 (5.9)	17 (14.9)	
Apex	15 (7.5)	2 (2.4)	13 (11.4)	
Hemodinamic imparment echo signs, *N* (%)	162 (81.4)	77 (90.6)	85 (74.6)	0.005
Causes of pericardial effusion				
Cancer, *N* (%)	67 (33.7)	22 (25.9)	45 (39.5)	0.064
Pericarditis, *N* (%)	27 (13.6)	6 (7.1)	21 (18.4)	0.035
Mechanical complications of AMI, *N* (%)	3 (1.5)	2 (2.4)	1 (0.9)	0.579
Autoimmune diseases, *N* (%)	6 (3.0)	1 (1.2)	5 (4.4)	0.242
RV heart failure, *N* (%)	1 (0.5)	–	1 (0.9)	0.999
Complications of cardiac surgery, *N* (%)	22 (11.1)	7 (8.2)	15 (13.2)	0.386
Complications of interventional cardiology, *N* (%)	42 (21.1)	36 (42.4)	6 (5.3)	<0.001
Anticoagulant overdose, *N* (%)	5 (2.5)	2 (2.4)	3 (2.6)	0.999
Other, *N* (%)	3 (1.5)	3 (3.5)	–	0.076
Unknown origin, *N* (%)	22 (11.1)	6 (7.1)	16 (14.0)	0.186
Setting of the procedure, *N* (%)				<0.001
*Bedside*	167 (83.9)	55 (64.7)	112 (98.2)	
*Cath lab*	32 (16.1)	30 (35.3)	2 (1.8)	
Imaging guidance, *N* (%)				<0.001
*Fluoroscopy*	24 (12.1)	23 (27.1)	1 (0.9)	
*Echo-assistance*	141 (70.9)	40 (47.1)	101 (88.6)	
*Echo-guidance*	13 (6.5)	1 (1.2)	12 (10.5)	
*Blind*	21 (10.6)	21 (24.7)	–	
Sedation/anesthesiologist support, *N* (%)	24 (12.1)	20 (23.5)	4 (3.5)	<0.001
Pericardial drainage, *N* (%)	163 (81.9)	72 (84.7)	91 (79.8)	0.485
Drained volume at first (ml), median (Q1–Q3)	577.5 (350.0–900.0)	460.0 (250.0–775.0)	630.0 (480.0–900.0)	0.003
Total drained volume (ml), median (Q1–Q3)	692.5 (487.5–1070.3)	650.0 (375.0–1110.0)	800.0 (585.0–1050.0)	0.061
Duration of drainage (h), median (Q1–Q3)	24.0 (20.3–42.8)	25.0 (20.0–44.0)	24.0 (21.0–42.0)	0.586

AMI, acute myocardial infarction; BP, blood pressure; CKD, chronic renal failure; COPD, chronic obstructive pulmonary disease; CVP, central venous pressure; HR, heart rate; LVEF, left ventricle ejection fraction; RV, right ventricle.

The majority of the pericardial effusions drained were classified as severe (>20 mm) and exhibited a circumferential distribution around the heart. Effusions primarily located anterior to the right ventricle were predominantly addressed via subxiphoid access (15.3%), while the apical route was preferentially utilized for effusions located at the level of the inferior and anterolateral walls (14.9%) and the true apex (11.4%).

Malignancy was the most prevalent etiology (33.7%, predominantly drained via the apical approach), followed by complications arising from interventional cardiology procedures (21.1%), most commonly electrophysiological such as catheter ablation of supraventricular or ventricular tachyarrhythmias or the implantation of pacemakers and cardiac defibrillators (see supplementary data). Acute or subacute pericarditis accounted for 13.6% of the cases (including one case of uremic pericarditis, one case of bacterial pericarditis with isolation of *Staphylococcus constellatus*, and one case of tuberculous pericarditis). Among the malignant causes (see supplementary data), lung cancer was the most frequent (49.3%), followed by leukemias and lymphomas (11.9%). Three cases were related to pericardial mesothelioma, while another three involved cardiac tumors, specifically originating from the left atrium. Complications of cardiac surgery represented 11.1% of the population. Finally, in 11.1% of cases, no definitive cause was identified.

Percutaneous punctures were predominantly conducted at the patient's bedside (83.9%), with US-guided or US-assisted procedures being the preferred method (77.4%). Nearly a quarter of the procedures conducted via the subxiphoid route were performed based on anatomical landmarks without any guiding imaging support (blind technique). A pericardial drain was placed in 81.9% of cases and was maintained for a median duration of 24 h (Q1–Q3 20.3–42.8).

Table 2 shows the primary outcomes observed during the follow-up period, which had a median duration of 17.2 months (Q1–Q3: 3.8–69.2). The median stay in the CICU was 48 h (Q1–Q3: 24.0–83.0), while the median overall hospital stay was 10 days (Q1–Q3: 6.0–15.5).

**Table 2:** Outcomes

	Total (*N* = 199)	Subxiphoid approach (*N* = 85, 42.7%)	Apical approach (*N* = 114, 57.3%)	*P*-value
Cardiac ICU stay (hours), median (Q1–Q3)	48.0 (24.0–83.0)	48.0 (24.0–72.0)	48.0 (24.0–88.5)	0.884
In-hospital stay (days), median (Q1–Q3)	10.0 (6.0–15.5)	9.0 (6.0–15.0)	10.0 (6.0–15.8)	0.806
Use of amines/inotropic drugs, *N* (%)	11 (5.5)	7 (8.2)	4 (3.5)	0.259
Success rate, *N* (%)	196 (98.5)	84 (98.8)	112 (98.2)	0.999
Major complications^a^, *N* (%)	1 (0.5)	1 (1.2)	–	–
Minor complications, *N* (%)	22 (11.1)	6 (7.1)	16 (14.0)	0.170
*Pneumothorax noticeable at the CXR*	4 (18.2)	1 (16.7)	3 (18.8)	
*Hepatic puncture – hemoperitoneum not requiring any intervention*	2 (9.1)	1 (16.7)	1 (6.2)	
*Transient cardiac puncture*	3 (13.6)	1 (16.7)	2 (12.5)	
*Pleuro-pericardial fistula*	13 (59.1)	3 (50.0)	10 (62.5)	
Pericarditic reaction, *N* (%)	16 (8.0)	10 (11.8)	6 (5.3)	0.160
Pleuritic reaction, *N* (%)	5 (2.5)	4 (4.7)	1 (0.9)	0.166
Pain in the puncture site, *N* (%)	44 (22.1)	25 (29.4)	19 (16.7)	0.049
Supraventricular arrhytmias, *N* (%)	20 (10.1)	9 (10.6)	11 (9.6)	0.999
NSVT, *N* (%)	4 (2.0)	3 (3.5)	1 (0.9)	0.315
Bradyarrhytmias, *N* (%)	12 (6.0)	6 (7.1)	6 (5.3)	0.765
Urgent cardiac surgery, *N* (%)	2 (1.0)	2 (2.4)	–	0.183
In-hospital death, *N* (%)	12 (6.0)	4 (4.7)	8 (7.0)	0.706
Total mortality, *N* (%)	19 (9.5)	5 (5.9)	14 (12.3)	0.202
Recurrence of pericardial effusion, *N* (%)	43 (21.6)	16 (18.8)	27 (23.7)	0.516
New pericardiocentesis, *N* (%)	30 (15.1)	11 (12.9)	19 (16.7)	0.550
Time from the previous procedure (days), median (Q1–Q3)	45.0 (17.8–137.3)	35.0 (17.5–75.5)	49.0 (20.0–180.0)	
Pericardial surgery, *N* (%)				0.999
*Pleuro-pericardial window*	5 (71.4)	2 (100.0)	3 (60.0)	
*Pericardiectomy*	1 (14.3)	–	1 (20.0)	
*Pericardio-peritoneal shunt*	1 (14.3)	–	1 (20.0)	

a Major complications referred to procedure-related events resulting in death or the need for serious medical intervention, including: laceration of the chambers, damage to the intercostal vessels, pneumothorax requiring thoracic drainage, sustained ventricular tachycardia, bacteremia likely related to the puncture.

CXR, chest X-ray; NSVT, nonsustained ventricular tachycardia.

The overall success rate reached 98.5%, with no significant differences observed between the two groups.

Ventricular perforation following a subxiphoid pericardiocentesis and requiring urgent cardiac surgery occurred only in one patient. This was the sole case of major complications recorded in the entire cohort, and represented also the only procedure-related death.

The overall incidence of minor complications was 11.1%, primarily attributed to the creation of a probable pleuro-pericardial fistula in the context of concomitant pleural effusion. This was resolved by evacuating the pericardial sac into the pleura, followed by subsequent thoracentesis to drain the mixed fluids.

No statistically significant differences emerged between the two approaches for either major or minor complications, nor for the occurrence of pericarditic-pleuritic reactions or nonsustained supraventricular or ventricular tachyarrhythmias. The incidence of pain at the puncture site was significantly higher in the group treated via subxiphoid access (29.4% vs. 16.7% in the apical group, *P* = 0.049), though it was generally self-limiting and managed with mild analgesic medications.

Recurrences of pericardial effusion were observed in 21.6% of cases, with 15.1% requiring a second procedure. The median time to the second procedure was 45 days (Q1–Q3: 17.8–137.3). Seven patients underwent nonemergent pericardial surgery due to the need for multiple and recurrent evacuative pericardiocenteses.

**Fig. 1:**
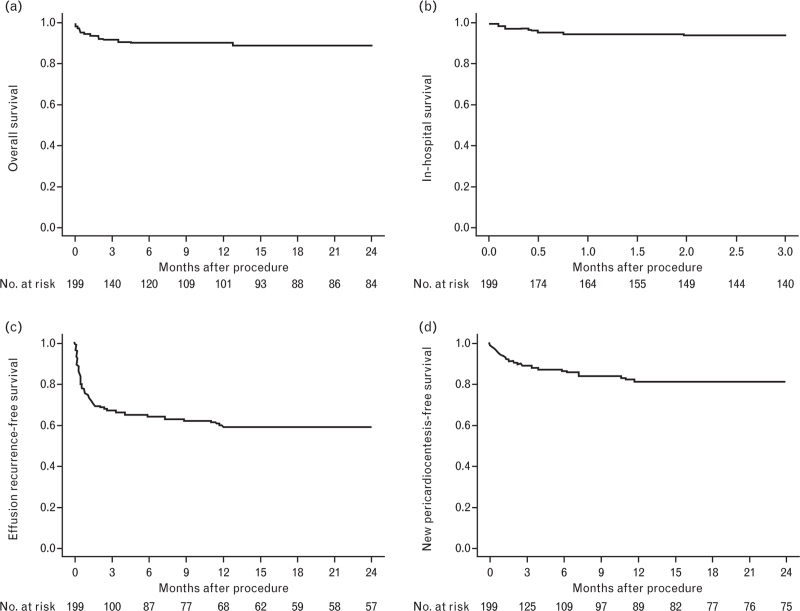
Kaplan–Meier curves to represent overall survival (panel a), in-hospital survival (panel b), effusion recurrence-free survival (panel c) and new pericardiocentesis-free survival (panel d) in the entire population.

Considering the postprocedural Kaplan–Meier survival curves estimated for the entire cohort, the overall survival, including all subsequent hospitalizations for any cause, was found to exceed 90% at 24 months following the procedure (Fig. 1, panel a). The probability of remaining free from recurrence of pericardial effusion was 60% at 1 year after the procedure (Fig. 1, panel c), while the likelihood of being free from subsequent pericardiocentesis remained above 80% at 24 months (Fig. 1, panel d).

**Fig. 2:**
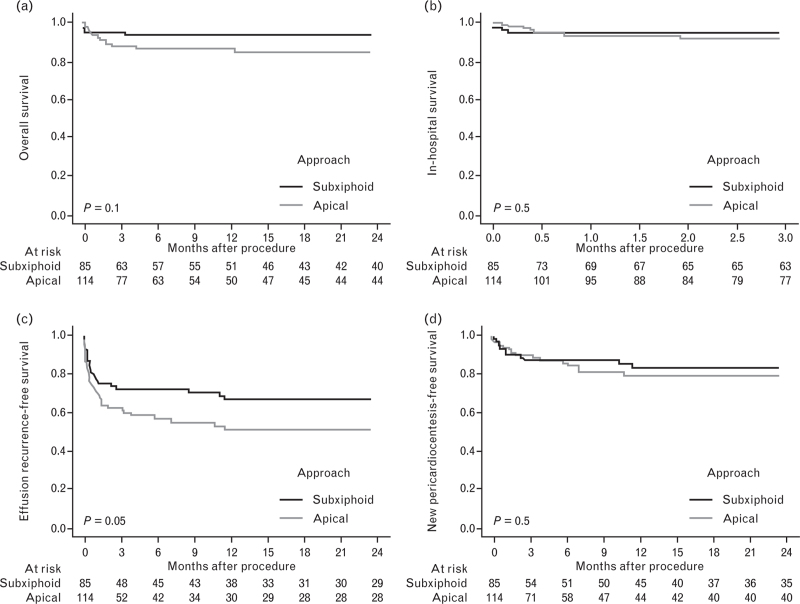
Kaplan–Meier curves to represent overall survival (panel a), in-hospital survival (panel b), effusion recurrence-free survival (panel c) and pericardiocentesis-free survival (panel d) stratified by approach type.

When comparing the two patient groups based on the type of pericardiocentesis performed, no statistically significant differences were observed in terms of overall survival, in-hospital survival, or the interval free from subsequent pericardiocentesis (Fig. 2, panels a, b, and d). However, patients who underwent pericardiocentesis via subxiphoid access demonstrated a statistically significant higher probability of surviving without recurrence of pericardial effusion (*P* = 0.05, Fig. 2, panel c).

## Discussion

Traditionally, most hospitals have favored the subxiphoid approach for percutaneous pericardiocentesis, as it is considered the safest method in the absence of imaging guidance.^4^ However, the efficacy and safety of US-guided or US-assisted procedures are well documented in the current literature, with failure rates ranging from 0.6 to 8.3%.^3,5–12^ Imaging support enhances safety, as the incidence of major complications in the largest observational studies of ultrasound-guided or fluoroscopic-guided pericardiocentesis is reported to be between 0.3% and 3.9%, while minor complications occur in 0.4% to 20% of cases.^3,7,9^

In our study cohort, the high success rate of 98.5% aligns with the findings of major publications. The primary factors contributing to this high success rate are undoubtedly the experience of the operators, along with the significant proportion of cases performed under echocardiographic guidance, suggesting that imaging support is a critical component for efficiently accessing the pericardial space.

Table 3 provides a comparative overview of the respective advantages and limitations associated with the apical and subxiphoid approaches to percutaneous pericardiocentesis.^2^

**Table 3:** Advantages and disadvantages of apical and subxiphoid approach in percutaneous pericardiocentesis

	Apical approach	Subxiphoid approach
Advantages	The increased thickness of the left ventricular wall enhances its capacity for self-sealing in the event of inadvertent puncture As ultrasound waves do not effectively penetrate air, echocardiographic guidance facilitates the avoidance of pulmonary structures The trajectory to the pericardial space is comparatively shorter	Reduced risk of pneumothorax
Disadvantages	Increased risk of ventricular puncture due to the close anatomical relationship with the left ventricle Heightened risk of pneumothorax owing to the proximity of the left pleural space	A steeper insertion angle may lead to inadvertent entry into the peritoneal cavity, whereas an overly medial trajectory increases the risk of right atrial puncture In certain cases, intentional traversal of the left hepatic lobe is performed when alternative access routes are unavailable The distance to the pericardial effusion is generally longer

Apical access was preferred in our center over the subxiphoid route (114 vs. 85 cases) – a proportion that contrasts with the most frequently selected technique according to the literature. Several potential advantages may arise from performing pericardiocentesis via the apical route. Generally, the apex offers the shortest path between the skin and the pericardial space, with minimal interference from lung parenchyma. Additionally, the US-guided method may assist in selecting the optimal needle entry site with greater accuracy.^6,13^

As previously mentioned, there is a lack of studies directly comparing the apical and subxiphoid approaches in the context of percutaneous pericardiocentesis. A recently published study^14^ analyzed the characteristics of 68 patients who underwent pericardiocentesis for both postsurgical and nonsurgical cardiac tamponades. According to the authors, apical access is more frequently employed than the subxiphoid approach in effusions occurring post cardiac surgery, given the predominant posterior-lateral and anterior-lateral localization of the fluid. In contrast, predominantly right ventricular effusions were more commonly observed in the nonsurgical group, although the frequency of both approaches was similar in this category (apical = 22, subxiphoid = 19). Another retrospective review,^15^ based on echocardiographic clips from 166 pericardial effusions, sought to evaluate the relationship between the distance from the skin surface to the pericardial fluid (ideally crossed by the needle) and the expected complications by different approaches, considering the interposed anatomical structures. This analysis suggested that the subxiphoid route not only resulted in a significantly longer pathway (mean distance of 56 mm, compared with 27 mm for the parasternal approach and 25 mm for the apical approach) but also led to a higher expected complication rate (79.7%, significantly greater than the 31.9% for the apical approach and 20.2% for parasternal visualization).

In our registry, the absence of significant differences between the two approaches indicates that, when performed by operators skilled in the maneuver, apical access is as effective and safe as the subxiphoid approach. The lack of any impact on short- to medium-term outcomes further supports this finding, as demonstrated by the nonsignificant results in the Kaplan–Meier analysis comparing overall survival, in-hospital survival, and survival free from new pericardiocentesis. The lower recurrence rate of pericardial effusion observed in the subxiphoid approach group (Fig. 2, curve 3, *P* = 0.05) may primarily be attributed to factors that are independent of the approach itself, such as the etiology of the pericardial effusion and the patient's clinical condition. Since most pericardiocentesis performed via the subxiphoid approach were conducted in the cath lab due to complications arising from interventional procedures (such as hemopericardium), the probability of recurrence was necessarily lower due to the prompt resolution of the iatrogenic causes. This contrasts with the recurrence rates associated with effusions stemming from subacute or chronic conditions, such as malignancy or systemic inflammatory diseases. Furthermore, the development of recurrence is influenced by the residual effusion size prior to catheter removal, as it is well established that the likelihood of a new pericardial effusion is significantly reduced when the drainage fully empties the pericardial space.^16,17^

This study is subject to several limitations. Firstly, as a retrospective analysis, the selection of the therapeutic intervention and the approach to the procedure were at the discretion of the operating physician. This decision, in various instances, may not have been determined exclusively by echocardiographic criteria, but could also have been partially influenced by the degree of familiarity each operator has with one procedure over another. Follow-up data, specifically regarding the recurrence of pericardial effusion, the need for new pericardiocentesis, and in-hospital mortality, were limited to those available within the digital archiving system of the hospital. This may have led to an underestimation of the actual mortality rate or recurrence of pericardial effusion. Furthermore, as previously discussed, the heterogeneity of the cohort is an additional limitation; the study did not differentiate based on factors such as the etiology of the effusion due to sample size constraints, even though these factors could potentially influence outcome differences. Lastly, the relatively low incidence of both major and minor complications prevented the robust identification of significant determinants influencing the outcomes.

## Conclusions

When performed by skilled personnel with imaging guidance, pericardiocentesis is a safe and effective procedure, essential in the urgent management of cardiac tamponade. Based on the findings of our study, the apical approach was demonstrated to be as effective and safe as the traditional subxiphoid access. These results reinforce expert recommendations to discontinue routine use of blind subxiphoid access, advocating for a more precise selection of the needle insertion site which should be guided by factors such as the location of the largest fluid collection, the shortest puncture path, and the preference and expertise of the operator with the approach.

## Acknowledgements

None.

### Conflicts of interest

There are no conflicts of interest.

## Supplementary Material
