# Supplementary material for: Efficacy and safety of apical access in percutaneous pericardiocentesis: a comparison with subxiphoid approach
Source: J Cardiovasc Med (Hagerstown). 2025 Aug 20;26(9):490–8. doi: 10.2459/JCM.0000000000001766 (PMC12721648; doi:10.2459/JCM.0000000000001766)
Supplement: Supplementary file 1 [file jcarm-26-490-s001.docx]

*Figure 3 - Distribution of complications of interventional procedures as a cause of pericardial effusion*

*
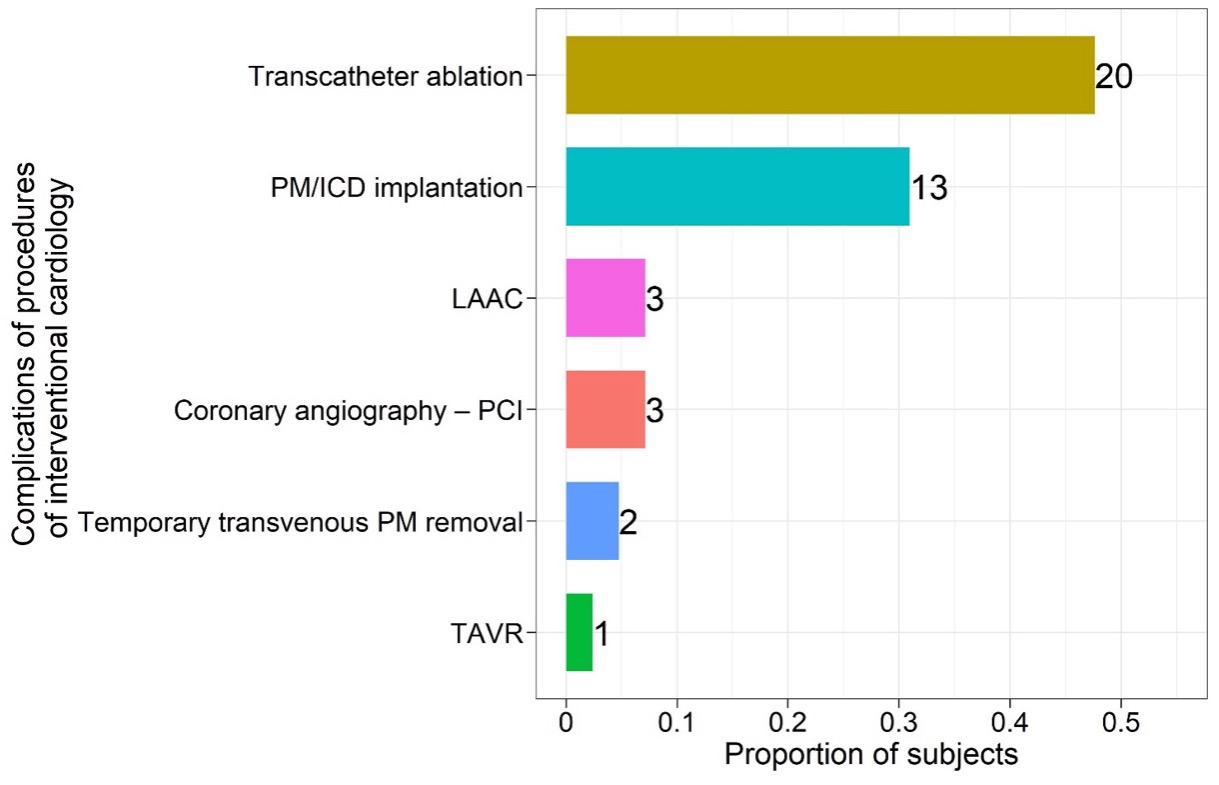
*

*Figure 4 - Distribution of primitive cancers as a cause of malignant pericardial effusion*


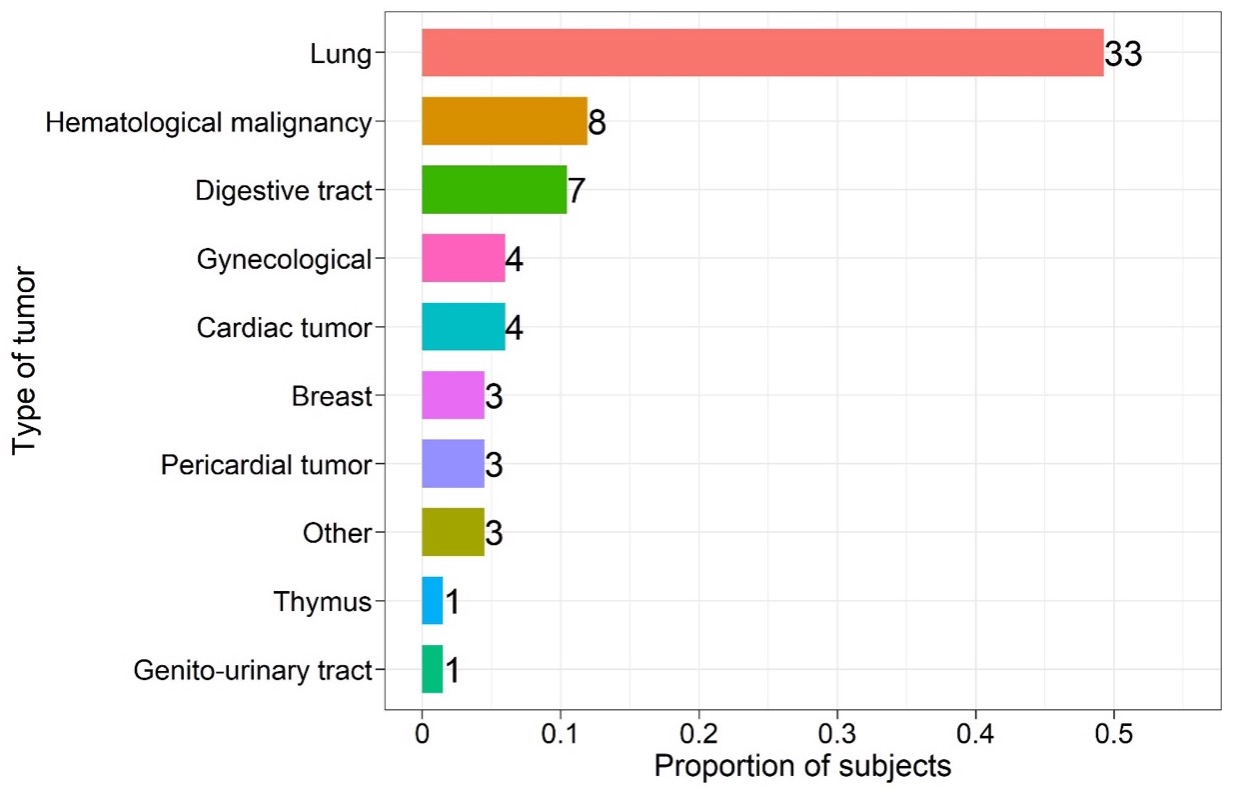


*Table 4 - Biochemical findings of serum and pericardial fluid*

|  | Total  (N=199) | Subxiphoid approach  (N=85, 42.7%) | Apical approach  (N=114, 57.3%) | p-value |
| --- | --- | --- | --- | --- |
| Seric hemoglobin (g/dL), median (Q1-Q3) | 11.1 (9.8-12.5) | 11.5 (10.1-12.9) | 10.9 (9.5-12.0) | 0.014 |
| Platelets (10^-6^/L), median (Q1-Q3) | 220.5 (165.3-318.5) | 211.5 (168.0-281.8) | 240.5 (164.5-328.3) | 0.218 |
| INR, median (Q1-Q3) | 1.21 (1.08-1.42) | 1.14 (1.06-1.34) | 1.26 (1.13-1.47) | 0.022 |
| CRP (mg/dL), median (Q1-Q3) | 4.7 (2.6-9.4) | 4.3 (2.0-10.3) | 5.2 (3.2-9.3) | 0.436 |
| Seric LDH (mU/mL), median (Q1-Q3) | 234.0 (198.0-324.0) | 231.0 (185.0-271.0) | 238.0 (207.3-337.0) | 0.132 |
| Serum proteins (g/dL), media (SD) | 6.05 (0.65) | 6.16 (0.73) | 6.01 (0.62) | 0.253 |
| Serum glucose (mg/dL), median (Q1-Q3) | 112.0 (95.0-138.0) | 107.0 (95.5-136.5) | 113.0 (95.0-139.0) | 0.503 |
| Drained material, N (%) |  |  |  | <0.001 |
| *Serous* | 40 (20.1) | 13 (15.3) | 27 (23.7) |  |
| *Sero-hematic* | 97 (48.7) | 30 (35.3) | 67 (58.8) |  |
| *Venous hematic* | 44 (22.1) | 28 (32.9) | 16 (14.0) |  |
| *Arterial hematic* | 17 (8.5) | 14 (16.5) | 3 (2.6) |  |
| *Purulent* | 1 (0.5) | - | 1 (0.9) |  |
| LDH pericardial fluid (mU/mL), median (Q1-Q3) | 638.0 (250.0-1378.5) | 479.0 (215.5-1149.0) | 655.0 (290.3-1465.0) | 0.335 |
| Proteins pericardial fluid (g/dL), media (SD) | 4.86 (0.89) | 4.99 (1.02) | 4.81 (0.84) | 0.311 |
| Glucose pericardial fluid (mg/dL), median (Q1-Q3) | 86.0 (62.0-112.0) | 93.0 (67.0-112.3) | 81.0 (58.0-110.0) | 0.441 |
| Exudate according to Light criteria, N (%) | 107 (98.2) | 31 (93.3) | 76 (100.0) | 0.165 |
| Citology positive for malignant cells, N (%) | 35 (19.6) | 9 (13.2) | 26 (23.4) | 0.136 |
| On the total of malignancies, N (%) | 35 (52.2) | 9 (40.9) | 26 (57.8) | 0.298 |

*INR = international normalized ratio; CRP = C-reactive protein; LDH = lactate dehydrogenase*

*Table 5 - Other clinical characteristics*

|  | Total  (N=199) | Subxiphoid approach  (N=85, 42.7%) | Apical approach  (N=114, 57.3%) | p-value |
| --- | --- | --- | --- | --- |
| Systolic BP (mmHg), median (Q1-Q3) | 116.0 (97.0-130.0) | 105.0 (80.0-120.0) | 120.0 (105.5-137.0) | <0.001 |
| Diastolic BP (mmHg), median (Q1-Q3) | 70.0 (60.0-80.0) | 70.0 (50.0-70.0) | 70.0 (60.0-80.0) | 0.006 |
| Mean BP (mmHg), median (Q1-Q3) | 84.5 (75.1-96.3) | 80.0 (58.8-87.9) | 88.3 (78.3-96.7) | <0.001 |
| HR (bpm), mean (SD) | 92.5 (19.8) | 94.9 (18.1) | 91.4 (20.5) | 0.336 |
| CVP (mmHg), mean (SD) | 16.1 (4.3) | 15.0 (4.9) | 16.6 (4.1) | 0.429 |
| Hypotension, N (%) | 82 (41.2) | 52 (61.2) | 30 (26.3) | <0.001 |
| Dispnoea, N (%) | 155 (77.9) | 62 (72.9) | 93 (81.6) | 0.201 |
| Antiplatelet therapy, N (%) | 27 (13.6) | 14 (16.5) | 13 (11.4) | 0.410 |
| Anticoagulant therapy, N (%) | 77 (38.7) | 34 (40.0) | 43 (37.7) | 0.857 |
| Post-procedurl parameters |  |  |  |  |
| Systolic BP (mmHg), median (Q1-Q3) | 120.0 (110.0-140.0) | 120.0 (110.0-130.0) | 123.0 (110.0-140.0) | 0.306 |
| Diastolic BP (mmHg), median (Q1-Q3) | 68.0 (60.0-80.0) | 68.5 (60.0-76.3) | 68.0 (60.0-80.0) | 0.991 |
| Mean BP (mmHg), median (Q1-Q3) | 85.0 (76.7-96.5) | 85.2 (76.5-94.1) | 85.0 (77.2-96.7) | 0.749 |
| HR (bpm), median (Q1-Q3) | 84.0 (72.0-95.0) | 82.0 (70.0-93.0) | 85.0 (75.0-95.0) | 0.177 |
